# Correction: GDP versus ESHAP Regimen in Relapsed and/or Refractory Hodgkin lymphoma: A Comparison Study

**Published:** 2015-10-01

**Authors:** Mani Ramzi, Aliraza Rezvani, Mehdi Dehghani

**Affiliations:** Department of Medical Oncology and Hematology and Bone Marrow Transplantation, Namazi Hospital, Shiraz University of Medical Sciences, Shiraz, Iran

## Notice of Republication

The article titled "GDP versus ESHAP Regimen in Relapsed and/or Refractory Hodgkin lymphoma: A Comparison Study"^1^ which was first published in *International Journal of Hematology-Oncology and Stem Cell Research*, Volume 9, Issue 1, pp. 10-14, has been republished on October 1, 2015, to correct some errors in the text as well as errors in the references. Please download this article again to view the correct version. The originally published, uncorrected article and the republished, corrected article are provided here for reference.

## Supporting Information

S1 File Originally published, uncorrected article. (PDF)

S2 File Republished, corrected article. (PDF)
