# Supplementary material for: Correction: GDP versus ESHAP Regimen in Relapsed and/or Refractory Hodgkin lymphoma: A Comparison Study
Source: Int J Hematol Oncol Stem Cell Res. 2015 Oct 1;9(4):218. (PMC4748694)
Supplement: S2 File — (PDF) [file IJHOSCR-9-218.s002.pdf]

IJHOSCR

Original Article

International Journal of Hematology- Oncology and Stem Cell Research

GDP versus ESHAP Regimen in Relapsed and/or Refractory Hodgkin lymphoma: A Comparison Study

Mani Ramzi, Aliraza Rezvani, Mehdi Dehghani

Department of Medical Oncology and Hematology and Bone Marrow Transplantation, Namazi Hospital, Shiraz University of Medical Sciences, Shiraz, Iran

Corresponding Author: Dr Aliraza Rezvani, Namazi Hospital, Department of Medical Oncology and Hematology and Bone Marrow Transplantation, Shiraz University of Medical Sciences, Zand Avenue, Shiraz, Iran
Tel & Fax: +987136474301
Email: ar.rezvani@hotmail.com

Received: 22, Aug, 2014
Accepted: 1, Nov, 2014

ABSTRACT

Background: Despite multiple published studies reporting result of salvage regimens for relapsed and refractory Hodgkin's lymphoma, there are no comparisons of different combinations.

Patients and methods: A total of 44 patients identified with refractory or relapsed Hodgkin's Lymphoma were considered eligible for this study. The Patients were randomly divided into two groups of 22, one of which were treated with GDP regimen (Gemcitabine, Dexamethasone and Cisplatin) and the other with EHSAP regimen (Etoposide, Methyl prednisolone, Cisplatin and Cytarabine) in a prospective manner. The results of each group were compared.

Results: There were 27.3% complete response, 31.8% more than 50% response, and 40.9% no response with GDP. ESHAP results were 29.5%, 24% and 45.5%, respectively.

Conclusion: There is no significant difference in response rate between GDP and ESHAP regimens as salvage chemotherapy in refractory or relapsed Hodgkin's Lymphoma.

Keywords: Hodgkin Lymphoma, Relapsed Hodgkin Lymphoma, Salvage Chemotherapy

INTRODUCTION

Treatment of limited stage of Hodgkin lymphoma by introducing new chemotherapy regimens and combining radiotherapy and chemotherapy has significantly evolved in recent years and failure in treatment is seen only in 10 to 20% of patients.[1,2]

Recently, there are tangible improvements in advanced stages of Hodgkin lymphoma but 10% of patients failed to achieve complete response with combined modality therapy and 20 to 40% of patients suffered from recurrence or progressive disease.[3,4]

Salvage chemotherapy and autologous bone marrow transplant has become the standard of treatment in recurrence or refractory Hodgkin's Lymphoma.[5,6] Different regimens of salvage chemotherapy have been introduced in literatures,[7-9] with the goal of attaining a higher response rate,[10,11] least side effects[12-14] and least damage to bone marrow cells in order to avoid distortion in next phase of treatment which needs mobilization and harvesting stem cells.[15-17] Considering acceptable progression free survival following this treatment, proper chemotherapy before autologous bone marrow transplant is a critical step.[18-20]

The two main salvage chemotherapies vastly implemented are GDP (Gemcitabine, Dexamethasone, and Cisplatine) with less hospitalization introduced by Baetz[21] and ESHAP (Etoposid, Methyl prednisolone, Cisplatine, Cytarabine) introduced by Aparicio et.al. The latter needs at least five days of hospitalization[22] and has

been used as the standard protocol in our Institute over the last few years.

Although several studies have been performed to demonstrate the efficacy of these protocols, no comparison has been made between these two treatment regimens.[23,24] In order to compare the efficacy of these two protocols, we conducted a randomized prospective clinical trial.

PATIENTS AND METHODS

Eligibility and evaluation of patients

The study included 50 patients with recurrent of Hodgkin Lymphoma between Jan 2010 and Dec 2011. All patients received standard protocol of ABVD (Doxorubicin, Bleomycin, Vinblastine and Dacarbazine) as the first -line of treatment.

Disease recurrence was histopathologically confirmed in patients with recurrence one year after their primary diagnosis, or there was radiologic evidence of recurrence in any organ in the body other than primary site. No histopathologic study was done on patients with recurrence disease in less than one years of diagnosis and radiologic evidence of recurrence in primary site. Other inclusion criteria were: age ≥ 16 years' old, Eastern cooperative oncology group performance states of 0-2, creatinine < 1.4 mg/dl, serum aspartate or alanine aminotransferase < 2.5 upper limit of normal and bilirubin < 1.5 ULN**.**

Patients with inclusion criteria were randomly assigned into two treatment groups: GDP and ESHAP (block randomization).

Complete physical examination, CBC with differentials and biochemistry profile (BUN, Cr and liver function tests) were conducted before starting treatment in each cycle and one week after chemotherapy.

This study was approved by the Institutional Review Board and Medical Ethics Committee of Shiraz University of Medical Science (SUMS). Meanwhile, all patients provided written informed consent.

Treatment plan

Chemotherapy in GDP group consisted of Gemcitabine 1000 mg/m2 on days 1 and 8; Dexamethasone 40 mg IV on days 1 to 4 and Cisplatin 75mg/m². In order to reduce the risk of Cisplatin-induced nephrotoxicity, patients were hospitalized for a maximum of 36 hours and hydrated with normal saline 12 hours prior to chemotherapy that was continued 8 hours after introduction of Cisplatin. Dexamethasone and Granisetron were used intravenously as anti-emetic agent. In ESHAP group, patients were hospitalized during chemotherapy and the protocol included the followings:

Etoposide 40 mg/m² on days 1 to 4, Methylprednisolone 500 mg IV on days 1 to 4, Cytarabine 2000 mg/m² on day 5 and Cisplatin 25 mg/m² on days 1 to 4. Courses were repeated every 3 weeks and anti-emetic agents used for treatment were the same as GDP protocol. Treatment cycles were delayed by 1 week for garnulocytopenia of < 1.0×10^9 or thrombocytopenia of < 100×10^9/L, or attenuation schedule was implanted.

Assessment of Treatment

All patients were evaluated during 3 weeks of third course of chemotherapy with complete physical examination and chest, abdomen and pelvis CT-scan. The primary objective of this study was to evaluate the response rate according to the National Comprehensive Cancer Network (NCCN) guide line version 1-2011.[25]

Chemotherapy side effects were evaluated based on National Cancer Institute Common Toxicity Criteria version 4.0.[26] PET scan or gadolinium scan was not done for patients.

RESULTS

A total of 44 patients who met the inclusion criteria were entered into the study. Mean age of patients was 29.73 (rang: 17- 56) in GDP group and 26.5 (rang: 18- 56) years old in EHSAP group. No statistically significant difference was observed between the two treatment groups (Mann-Whitney test, *P* value: 0.655).

In GDP group, disease stage was stage II in 45.5%, stage III in 45.5% and stage IV in 9% of patients. In ESHAP group, these proportions were 54.6%, 22.7% and 22.7%, respectively, suggesting the equal distribution of patients in both groups with respect to disease stage. In GDP group, 77% of patients had

first relapse, 9.1% had second relapse and 13.6% of them had primary refractory disease. In ESHAP group, these values were 72.2%, 4.5% and 22.7%, respectively (Fisher's exact test, *P*-value: 0.546). Therefore, No difference was observed in disease stages between two groups.

The mean time to relapse was 20.42 and 16.35 months in GDP and ESHAP groups, respectively and there was no statistically significant difference between the two groups (Mann-Whitney significance: 0.247).

Considering the aforementioned factors, it can be concluded that patients were equally distributed in both groups with respect to age, sex, stage and time to relapse.

27.3% of patients in GDP group had complete response, 31.8% had more than 50% response and 40.9% had no response. The results in ESHAP group were 31.8%, 18.2% and 50%, respectively. Statistical analysis with the Chi-square test showed that response rate was identical in both groups (significance: 0.578). Overall response rate in GDP and ESHAP groups was 54.1% and 50%, respectively (significance: 0.763).

None of the patients with recurrent stage IV disease in both groups reached complete response. Also, none of patients with primary refractory disease had response to used protocols but responded to IEV (Ifosfamide, Epirubicin and Etoposide).

Treatment-related Toxicity

Of two patients with thrombocytopenia in GDP group, one (4.5%) developed grade I and one (4.5%) developed grade III. This event in ESHAP group was seen in 9.1 % (grade II).

In GDP group, grade III neutropenia was seen in one (4.5%) patient and two (9.1%) patients experienced grade II neutropenia in ESHAP group.

Creatinine in ESHAP group was raised from 1.8 to 2 mg/dl in one patient and remained in this level after one-year follow-up period. AST and ALT also rose to greater than 2 times about 2x ULN (upper normal limit) during chemotherapy and returned to normal levels after two weeks in this patient.

In GDP group, one patient suffered from hyperglycemia in the last cycle of treatment and blood sugar was not corrected after termination of treatment.

DISCUSSION

Because achievement to adequate response to salvage chemotherapy before bone morrow transplantation is important which influences transplantation's results, in this study the researcher attempted to compare the results of two method of treatment: ESHAP vs. GDP. The former has been used as a common treatment regimen at our center over the last few years and needs 5 days of hospitalization. The later method which has acceptable effectiveness with far less side effects and required hospitalization i.e. GDP. Baetz[21] introduced GDP protocol (Gemcitabine, Dexamethasone and Cisplatin) and evaluated patients after 2 cycles of chemotherapy. Among his patients, 4 had complete response, 12 had partial response and 7 had stable disease (without progression on treatment). In the study conducted by Aparicio,[22] 22 patients were treated with ESHAP (Etoposid, Methyl prednisolone, Cisplatin and Cytarabine) protocol and were evaluated after 3 cycles of chemotherapy. At the end of the study, 9 patients had complete response and 5 patients had partial response (overall response: 73%).

Chemotherapy regimen in GDP group of our study was slightly different from Baetz study.[21] In this study, patients were hospitalized at least 36 hours in order to adequate hydration and reduce the risk of Cisplatin-related nephrotoxicity, while in Baetz study[21] this protocol was prescribed as outpatient and manitol was used in addition to dextrose/saline before Cisplatin. Like Baetz study, Nephrotoxicity was not seen in our patients in GDP group.

Due to lack of response to initial salvage treatment protocol (GDP or EHSAP); cross-over trial was performed in three patients in two groups at the beginning of our study. None of these patients showed response to second-line salvage chemotherapy (either GDP or ESHAP), therefore, this trend was stopped.

In Baetz study,[21] 52% of patients treated with GDP protocol had stage III and IV diseases and 48% had stage I and II. In the current study, these rates were 45.5% and 54.5%, respectively.

In this study, 13.6% of patients in GDP group had primary refractory disease, while only 26% of patients had primary refractory disease in Baetz study.[21] Compared to Baetz study in which patients received 2 cycles of chemotherapy, in this study patients received 3 cycles of chemotherapy.[21] In the current study, overall response rate in GDP group was 54.1%, while it was reported 69.5% in Baetz study.[21]

In the present study, overall response rate was 50% in ESHAP group, while it was 73% in patients treated with similar protocol in Aparicio study.[22]

In Aparicio study,[22] 59% of patients developed myelotoxicity (grades III and IV) and one patient died of neutropenic fever, while in the present study side effects were significantly lower and there were no mortality.

In Baetz study,[21] four patients needed hospitalization, 8.6% were reported to suffer from neutropenia (grade III) and 13% developed thrombocytopenia. In the present study, 4.5% of patients showed grade III neutropenia and 4.5% of patients showed grade I and III thrombocytopenia. Meanwhile, none of the patients needed hospitalization.

On this study, there was no significant difference in response rate between the two salvage regimens, but GDP regimen can be used as an outpatient regimen with low toxicity. Based on this study, these two regimens were not suitable options for primary refractory Hodgkin lymphoma as salvage treatment.

CONCLUSION

There is no significant difference in overall response rates between the two protocols but due to least toxicity and lower health care costs result from less hospitalization, GDP could be considered as the better option for salvage regimen.

AKNOWLEDGEMENT

This study was supported by Shiraz University of Medical Sciences (proposal No: 90-5788) and based on Dr.Rezvani’s fellowship thesis.

CONFLICT OF INTEREST

The authors have declared no conflicts of interest.

REFERENCES

1. Armitage JO. Early-stage Hodgkin´s lymphoma. N Engl J Med. 2010; 363(7): 653-662.
2. Straus DJ, Portlock CS, Qin J, et al. Results of a prospective randomized clinical trial of doxorubicin, bleomycin, vinblastine and dacarbazine (ABVD) followed by radiation therapy(RT) versus ABVD alone for stage I, II and IIIA non bulky Hodgkin disease. Blood. 2004; 104: 3483-3489.
3. Duggan DB, Petroni GR, Johnson JL, et al. Randomized Comparison of ABVD and MOPP /ABV Hybrid for the treatment of advanced Hodgkin ׳s disease: Report of an Intergroup Trial. J Clin Oncol. 2003; 21: 607-614.
4. Kuruvilla J. Standard therapy of advanced Hodgkin lymphoma. Hematology Am Soc Hematol Educ Program. 2009; 497-506.
5. Lazarous HM, Rowlings PA, Zhang MJ, et al. Auto transplants for Hodgkin´s disease in patients never achieving remission: a report from the autologous Blood and Marrow Transplant Registry. J Clin Oncol. 1999; 17(2): 534-545.
6. Andre´ M, Henry-Amar M, Pico J-L, et al. Comparison of high-dose therapy and autologous stem-cell transplantation with conventional therapy for Hodgkin’s disease induction failure: a case-control study. J Clin Oncol. 1999; 17(1):222-229.
7. Bonfante V, Viviani S, Santoro A, et al. Ifosfamide and vinorelbine: an active regimen for patients with relapsed or refractory Hodgkin’s disease. Br J Haematol. 1998; 103(2):533-535.
8. Ferme´ C, Mounier N, Divine M, et al. Intensive salvage therapy with high-dose chemotherapy for patients with advanced Hodgkin’s disease in relapse or failure after initial chemotherapy: results of the Groupe d’Etudes des Lymphomes de l’Adulte H89 Trial. J Clin Oncol. 2002; 20(2):467-475.
9. Ribrag V, Nasr F, Bouhris JH, et al. VIP (etoposide, ifosfamide and cisplatinum) as a salvage intensification program in relapsed or refractory Hodgkin’s disease. Bone Marrow Transplant. 1998; 21(10): 969-974.
10. Colwill R, Crump M, Couture F, et al. Mini-BEAM as salvage therapy for relapsed or refractory Hodgkin’s disease before intensive therapy and autologous Bone Marrow Transplantation. J Clin Oncol. 1995; 13(2):396-402.

11. Pfreundschuh MG, Rueffer U, Lathan B, et al. Dexa-BEAM in patients with Hodgkin's disease refractory to multidrug chemotherapy regimens: a trial of the German Hodgkin's Disease Study Group. J Clin Oncol. 1994; 12(3):580-586.
12. Rodriguez J, Rodriguez MA, Fayad L, et al. ASHAP: a regimen for cyto reduction of refractory or recurrent Hodgkin's disease. Blood. 1999; 93(11):3632-3636.
13. Chau I, Harries M, Cunningham D, et al. Gemcitabine, cisplatin and methylprednisolone chemotherapy (GEM-P) is an effective regimen in patients with poor prognostic primary progressive or multiply relapsed Hodgkin's and non-Hodgkin's lymphoma .Br J Haematol. 2003; 120(6):970-977.
14. Josting A, Rudolph C, Reiser M, et al. Time intensified dexamethasone/cisplatin/cytarabine: an effective Salvage therapy with low toxicity in patients with relapsed and refractory Hodgkin's disease. Ann Oncol. 2002; 13(10):1628-1635.
15. Proctor SJ, Jackson GH, Lennard A, et al. Strategic approach to the management of Hodgkin's disease incorporating salvage therapy with high dose ifosfamide, etoposide and epirubicin: a Northern Region Lymphoma Group study (UK). Ann Oncol. 2003; 14 (suppl 1):I47-50.
16. Bartlett NL, Niedzwiecki D, Johnson JL, et al. Gemcitabine, vinorelbine, and pegylated liposomal doxorubicin (GVD), a salvage regimen in relapsed Hodgkin's lymphoma: CALGB 59804. Ann Oncol. 2007; 18(6): 1071-1079.
17. Bonfante V, Viviani S, Devizzi L, et al. High-dose ifosfamide and vinorelbine as salvage therapy for Relapsed or refractory Hodgkin's disease. Eur J Haematol Suppl. 2001; 64:51-55.
18. Moskowitz CH, Kewalramani T, Nimer SD, et al. Effectiveness of high dose chemo radiotherapy and autologous stem cell transplantation for patients with biopsy-proven primary refractory Hodgkin's disease. Br J Haematol. 2004; 124:645-652.
19. Sirohi B, Cunningham D, Norman RA, et al. Long-term outcome of high dose chemotherapy and autologous stem cell transplantation in relapsed/refractory Hodgkin's disease: A cohort of 199 pts from Royal Mardsen Hospital. J Clin Oncol. 2006;24(suppl):7622
20. Crump M, Smith AM, Brandwein J, et al. High dose etoposide and melphalan and autologous bone marrow transplantation for patients with advanced Hodgkin's disease: importance of disease status at transplant. J Clin Oncol. 1993; 11(4):704-711.
21. Baetz T, Belch A, Couban S, et al. Gemcitabine, dexamethasone and cisplatin is an active and non-toxic chemotherapy regimen in relapsed or refractory Hodgkin's disease: a phase II study by the National Cancer Institute of Canada Clinical Trials Group. Ann Oncol. 2003; 14(12):1762-1767.
22. Aparicio J, Segura A, Garcera S, et al. ESHAP is an active regimen for relapsing Hodgkin disease .Ann Oncol. 1999; 10:593-595.
23. Kuruvilla J, Keating A, Crump M. How I treat relapsed and refractory Hodgkin lymphoma. Blood. 2011 Apr 21; 117(16): 4208-4217.
24. Collins GP, Parker AN, Pocock C, et al. Guideline on the management of primary resistant and relapsed classical Hodgkin lymphoma .Br J Haematol. 2013 Oct 1. doi: 10.1111/bjh.12582. [Epub ahead of print]
25. National comprehensive Cancer Network V1.2011 guideline on Hodgkin lymphoma [Cited 2012 Nov1]; available from http://www.nccn.org/ professionals/physician_gls/f_guidelines.asp
26. Common Terminology Criteria for adverse events (CTCAE) Version 4.0. [Internet], 2010 June 14. [2012 nov 14]. Available from: http: //evs.nci.nih.gov/ftp1/CTCAE/CTCAE_4.03_2010-06-14_QuickReference_5x7.pdf
